# NAV2 positively modulates inflammatory response of fibroblast‐like synoviocytes through activating Wnt/β‐catenin signaling pathway in rheumatoid arthritis

**DOI:** 10.1002/ctm2.376

**Published:** 2021-05-01

**Authors:** Ran Wang, Meng Li, Weijun Wu, Yuanye Qiu, Wei Hu, Zhaoyi Li, Zhou Wang, Yue Yu, Junyi Liao, Wuyi Sun, Jianchun Mao, Yi Zhun Zhu

**Affiliations:** ^1^ State Key Laboratory of Quality Research in Chinese Medicine, School of Pharmacy Macau University of Science and Technology Macau China; ^2^ Department of Rheumatology Longhua Hospital of Shanghai University of Traditional Chinese Medicine Shanghai China; ^3^ Department of Pharmacology, Shanghai Key Laboratory of Bioactive Small Molecules, School of Pharmacy Fudan University Shanghai China

Dear Editor,

Rheumatoid arthritis (RA) is a common chronic autoimmune disease that causes progressive joint destruction and involves severe damage to physical function and life quality.^1^ Without rational drug intervention, 80% patients will become incapable of mobility after 3 years of illness.^2^ In the pathogenesis of RA, activated fibroblast‐like synoviocytes (FLS) participate in the inflammatory process of RA through their own proliferation, migration, and invasion.^3^, ^4^ However, despite the thorough research, there are still gaps in our understanding of RA pathogenesis. Therefore, it is important to better understand the etiopathogenesis of RA.

Neuron navigator 2 (NAV2) belongs to the neuron navigator family which mainly regulates growth, migration, and regeneration of neurons in the nervous system and is highly expressed in the brain, kidney, liver, and also existed in the skeleton, heart, placenta, as well as abundantly elevated in colon cancers.^5^ Previous studies have found that NAV2 facilitates invasion of cutaneous melanoma cells and plays a vital role in the poor prognosis of melanoma patients.^6^ Also, in the results of proteomics, NAV2 is abnormally highly expressed in RA patients’ primary FLS compared with healthy volunteers.^7^ However, the specific role of NAV2 in RA remains unknown.

We first examined NAV2 protein level in blood samples and found a significant increase of NAV2 expression in RA patients compared with osteoarthritis (OA) patients or healthy samples (Figure 1A). The clinically relevant information and spreadsheet are shown in Table S1. Then we performed an immunofluorescence double‐staining experiment. The expression of vimentin indicated that the cells derived from synovium tissues were primary FLS and NAV2 expression was significantly upregulated (Figure 1B). Moreover, we confirmed the significant elevation of NAV2 in the primary RA synovial cells by using RT‐qPCR and Western blot analysis (Figure 1C). Then we observed and validated NAV2 expression in AIA rat synovial tissues. Images of rats’ hind paws showed significant exacerbation in inflammation and soft tissue swelling. Micro‐CT and histological analysis presented swollen joints, bone destruction, synovial membrane hyperplasia accompanying serious inflammatory cell infiltration, and joint tissues with pannus formation (Figure S1A). The model group also developed much more severity and higher incidence of arthritis as determined by arthritis scores and hind paw volumes compared to the normal group, and the mean body weights of rats in the two groups increased during the experiment (Figure S1B). We furthermore monitored the production of proinflammatory cytokines IL‐Iβ, IL‐6, and TNF‐α in the serum by ELISA kit, as shown in Figure S2A, the levels of IL‐Iβ, IL‐6 and TNF‐α were dramatically increased. Additionally, the protein expression of MMP‐9, COX‐2, and IL‐6 in inflamed joints was significantly upregulated (Figure S2B), indicating that the AIA model has been successfully established. Intriguingly, expression of NAV2 markedly increased both on the protein level and mRNA level (Figure 1D). And we could see the average integrated optical density (IOD) of NAV2 protein was significantly higher in the ankle joint of AIA rats (Figure 1E).

**FIGURE 1:**
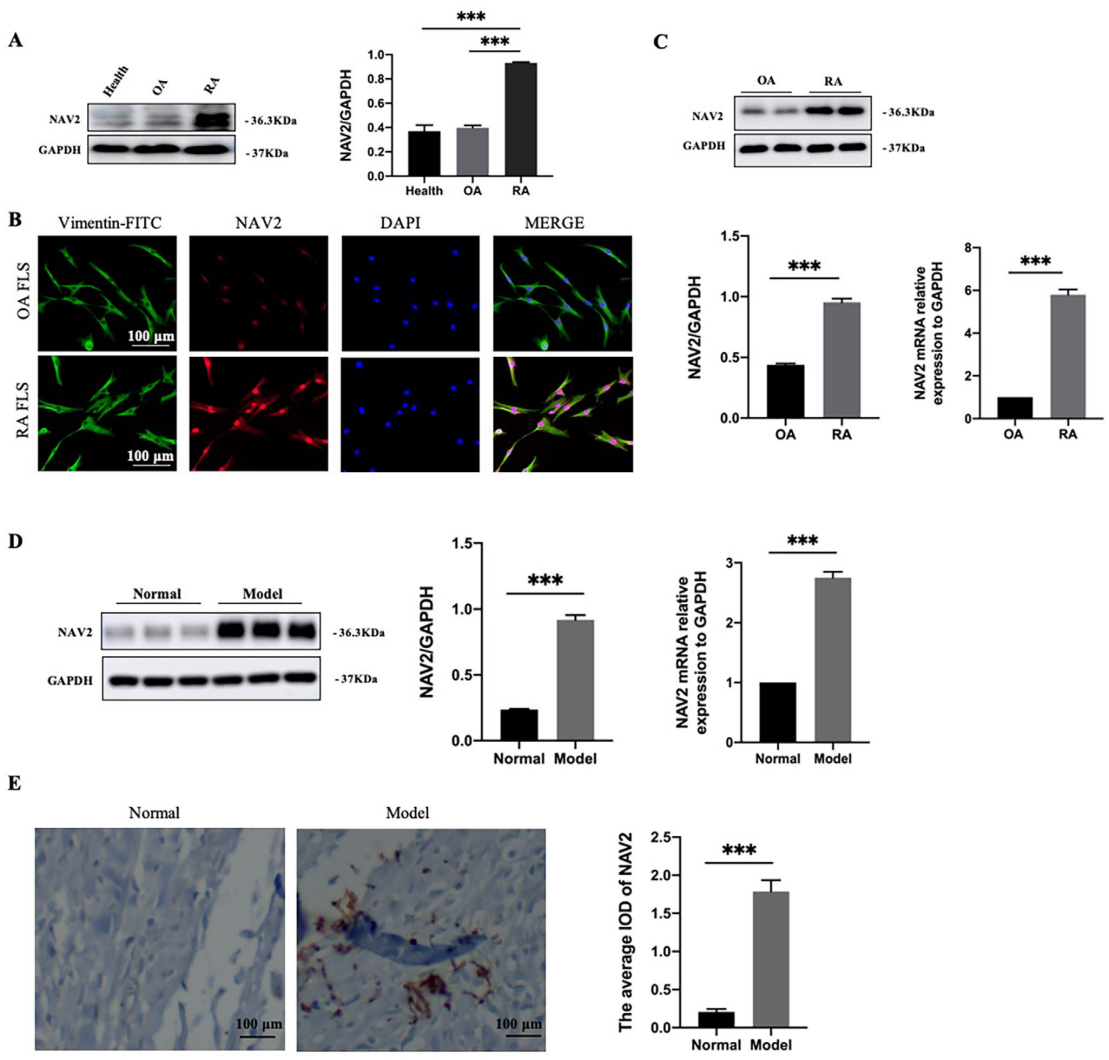
NAV2 expression is increased in blood samples and FLS from RA patients as well as in AIA rat synovium. (A) NAV2 expression in blood samples from healthy volunteers (*n* = 6) and patients with RA (*n* = 20) or OA (*n* = 9). Primary synovial cells extract from OA and RA patients’ synovial tissues. (B) The expression of NAV2 and vimentin were analyzed by immunofluorescence double‐staining analysis. DAPI was used to stain nuclei (blue). Vimentin and NAV2 were used to stain vimentin (green) and NAV2 (red). (C) The expression of NAV2 mRNA and protein in FLS were determined. (D) Western blot result and RT‐qPCR analysis of NAV2 expression in synovial tissues from AIA rats were examined; GAPDH was used as a loading control. (E) Immunohistochemistry staining of NAV2 in ankle joint. Data are means ± SEM from at least three independent experiments. *n* = 6–8 rats per group. Scale bars indicate 100 μm for H&E staining. Scale bars indicate 50 μm for immunohistochemistry and immunofluorescence staining. *** *p* < 0.001

In vitro assay, first, we used MH7A cells treated with TNF‐α (20 ng/ml) for 0, 3, 6, 12, 24 and 48 h to evaluate an inflammatory response (Figure S3A‐G). Interestingly, *NAV2* mRNA level reached a peak at 3 h and then declined, NAV2 protein level showed a continuous increase from 0 to 24 h and decreased slightly at 48 h (Figure 2A). After transfection with siRNA to knockdown NAV2 expression in MH7A cells, we could see that siRNA downregulated NAV2 mRNA and protein expression when compared with scrambled control siRNA (si Scr) (Figure 2B). Intriguingly, accompanied by a decrease of NAV2, the protein and mRNA expression of IL‐6, MMP‐9, and COX‐2 were also decreased (Figure 2C‐E). Besides, silencing NAV2 significantly decreased protein expression of ICAM‐1 and VCAM‐1 (Figure 2F and G). Also, the mRNA level of *IL‐8* declined after NAV2 knockdown (Figure 2H). More importantly, lower NAV2 expression led to the decline of IL‐1β and IL‐6 secretion in the supernatants of the TNF‐α‐stimulated MH7A cells (Figure 2I and J). Immunofluorescence double staining also displayed that the co‐expressions of NAV2 and COX‐2 upon TNF‐α induction disappeared when NAV2 was knocked down (Figure 2K). As Wnt/β‐catenin pathway was stimulated in AIA rats (Figure S2C), the protein levels of GSK‐3β, β‐catenin, c‐myc, CyclinD1, and MMP‐3 appeared to be markedly attenuated when NAV2 was silenced (Figure 2L). Furthermore, silenced NAV2 expression significantly impeded cell motility, migration and invasive capabilities. Cell growth rates in si *NAV2*‐transfected cells were also markedly diminished (Figure 2 M).

**FIGURE 2:**
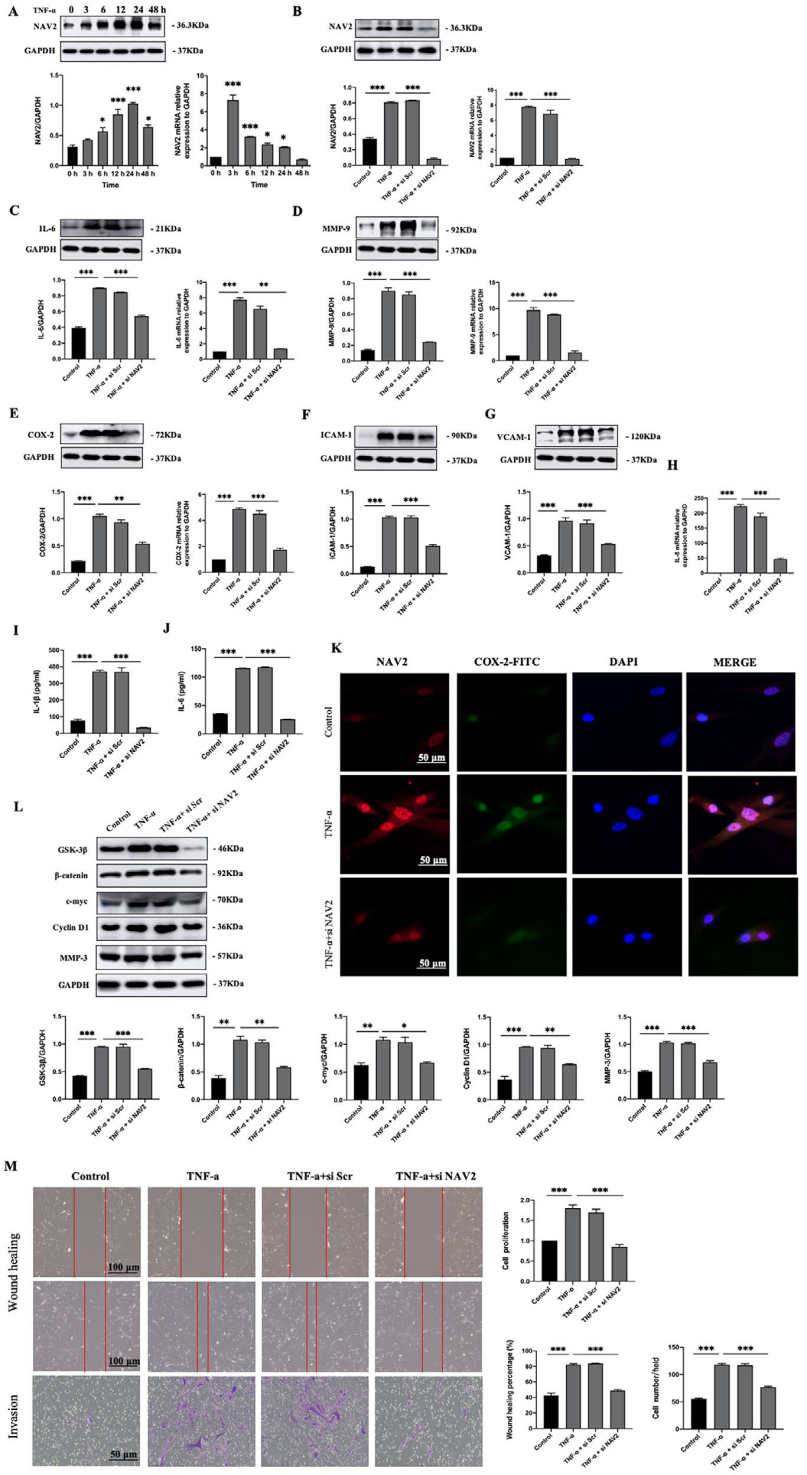
NAV2 positively regulates the inflammatory response and promotes migration, invasion and proliferation in MH7A cells through activating the Wnt/β‐catenin pathway. (A) The expression level of NAV2 in MH7A cells, at the different times following TNF‐α treatment, was determined by Western blot mothed and RT‐qPCR analyses, GAPDH was used as loading control. (B‐E) MH7A cells were transfected with si Scr or si *NAV2* prior to treatment with TNF‐α (20 ng/ml), the mRNA expression levels of *NAV2*, *COX‐2*, *IL‐6*, and *MMP‐9* were measured by RT‐qPCR after stimulation for 3 h, the protein expression levels were assessed by Western blot after stimulation for 12 h. (F, G) NAV2 silencing resulted in decreased protein expression of ICAM‐1 and VCAM‐1 after TNF‐α stimulation for 12 h. (H) The mRNA level of *IL‐8* decreased after silencing NAV2. (I, J) IL‐1β and IL‐6 production, in the culture supernatants, were assessed by ELISA after knockdown NAV2. (K) Cells were subjected to immunofluorescence staining for NAV2 and COX‐2. DAPI was used to stain nuclei (blue). NAV2 and COX2 pAb were used to stain NAV2 (red) and COX‐2 (green); Scale bars, 50 μm. (L) MH7A cells were transfected with si Scr or si *NAV2* for 48 h and then stimulated with TNF‐α for 12 h, representative immunoblots examining GSK‐3β, β‐catenin, c‐myc, CyclinD1 and MMP‐3 protein expression were detected by Western blot. (M) Wound healing (scale bars, 100 μm) and transwell invasion assay (scale bars, 50 μm) showed the capacity of horizontal migration and vertical migration, CCK‐8 showed the proliferation results. Data are presented as mean ± SEM of more than three independent experiments. *p < 0.05, **p < 0.01, ***p < 0.001

We next investigated the underlying mechanisms by which NAV2 promoted inflammatory response in RA. We found that NAV2 could be regulated by transcription factor E2F1. First, we found E2F1 increased in RA FLS and AIA rat synovial tissues (Figure 3A and B), as well as in MH7A cells subjected to TNF‐α for indicated times (Figure 3C). Then overexpression of E2F1 promoted NAV2 protein level, while knockdown of E2F1 by siRNA inhibited NAV2 expression. Also, we found that overexpression of E2F1 upregulated MMP‐3 and MMP‐9 expression, which are the downstream proteins in Wnt/β‐catenin signaling pathway and related with invasion and proliferation in RA, and also increased phenotype protein levels of VCAM‐1 and ICAM‐1 significantly. Whereas knockdown of E2F1 produced the opposite effect (Figure 3D‐O). Immunofluorescence double analysis confirmed that treatment with si *E2F1* also resulted in decreased E2F1 and NAV2 significantly (Figure 3P). Furthermore, we found that elevated E2F1 expression could enhance NAV2 transcription level dramatically by luciferase reporter assay (Figure 3Q). Then the CHIP‐qPCR results indicated that E2F1 could bind to the promoter region of NAV2 from ‐2000 to +500 (Figure 3R and S).

**FIGURE 3:**
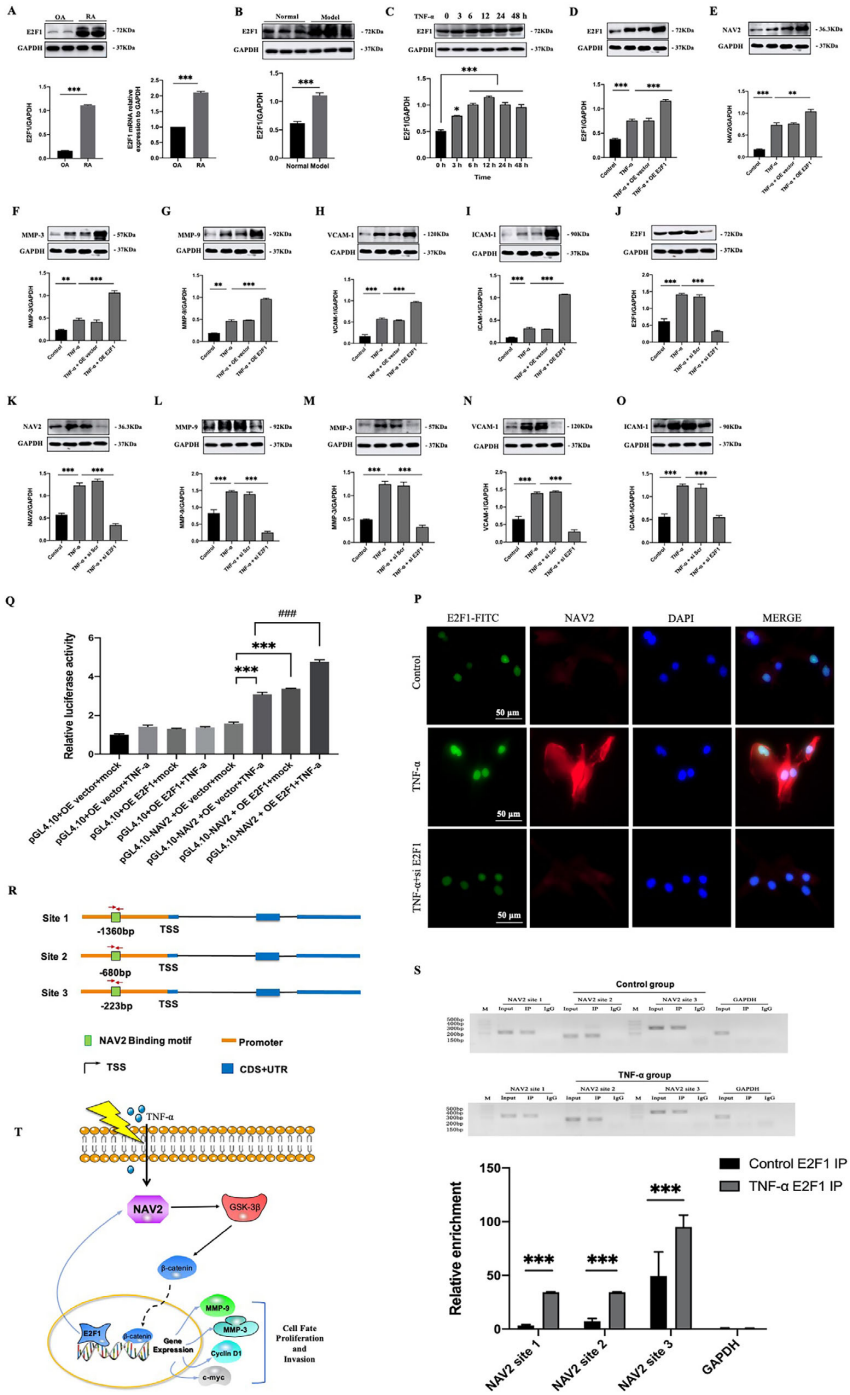
Transcription factor E2F1 binds to NAV2 promoter to regulate NAV2 expression. (A) The expression of E2F1 mRNA and protein in RA FLS were determined. (B) Western blot result and quantitative analysis of E2F1 expression in synovial tissues from AIA rats. (C) E2F1 was upregulated in TNF‐α‐induced MH7A cells inflammation. MH7A cells were treated with TNF‐α (20 ng/ml) for indicated times, using Western blot to determine E2F1 protein expression. (D–I) The expression of E2F1 was validated in MH7A cells transfected with E2F1‐cDNA (OE E2F1) or control empty vector (OE vector). Overexpression (OE) of E2F1 was confirmed by Western blot and NAV2, MMP‐3, MMP‐9, VCAM‐1, and ICAM‐1 level increased in E2F1 overexpressing cells, all blots shown are representative images from at least three replicates. (J–O) E2F1 knockdown (si *E2F1*) reversed the Wnt/β‐catenin signaling pathway and phenotypes in TNF‐α‐induced MH7A cells. (P) Cells were subjected to immunofluorescence staining for E2F1 and NAV2. DAPI was used to stain nuclei (blue). E2F1 and NAV2 pAb were used to stain NAV2 (red) and E2F1 (green); Scale bars, 50 μm. (Q) Effect of E2F1 on NAV2 gene promoter activity. Luciferase assay was performed to analyze NAV2 dependent transcriptional activity by using 293T cells. Data are presented as mean ± S.D, *n* = 3, ***p < 0.001 vs OE vector+mock group, ^###^ *p* < 0.001 vs OE vector+TNF‐α group. (R) Schematic diagram of three pairs of primers designed for CHIP spanning the NAV2 promoter. (S) E2F1 bound to the NAV2 promoter indicated by CHIP in MH7A cells. IgG from rabbit served as a control. (T) Working model for a critical role of NAV2 mediating inflammatory response in TNF‐α‐induced MH7A cells. MH7A cells have a higher expression level of NAV2 after TNF‐α‐induced as well as AIA rats. Increased NAV2 is regulated by transcription factor E2F1 and then activates the Wnt/β‐catenin signaling pathway, and subsequently causing inflammatory response in RA. Data are expressed as means ± SEM of three independent experiments. **p < 0.01, ***p < 0.001

In conclusion, the present study revealed an important novel finding that NAV2 plays a critical role in RA, and provided a new understanding of the molecular mechanism of RA which is unique and previously unreported. We also speculate targeting NAV2 might not just affect inflammation in RA but could also interfere with a major cell‐cell interaction involved in sensitization of joint‐innervating neurons that drive pain in arthritis.^8^ Therefore, NAV2 provides an attractive novel target to intervene inflammatory diseases, especially RA.

## AUTHOR CONTRIBUTIONS

R. Wang, J. Mao, and Y. Zhu designed research, analyzed data, and wrote the manuscript; and R. Wang, M. Li, W. Wu, Y. Qiu, W. Hu, Z. Li, Z. Wang, Y. Yu, J. Liao, W. Su performed research. All authors read and approved the manuscript.

## ETHICS APPROVAL AND CONSENT TO PARTICIPATE

All the experimental processes were conducted within the approved guidelines of the Ethics Review Committee for Animal Research of Macau University of Science & Technology. Appropriate measures were taken to minimize the use of animals as well as their suffering.

## CONFLICT OF INTEREST

The authors declare that they have no conflict of interest.

## Supporting information

Supporting Information

## References

[1] Sparks JA . Rheumatoid arthritis. Ann Intern Med. 2019;170(1):ITC1‐ITC16. 30596879 10.7326/AITC201901010

[2] Aletaha D , Smolen JS . Diagnosis and management of rheumatoid arthritis: a review. JAMA. 2018;320(13):1360‐1372. 30285183 10.1001/jama.2018.13103

[3] Bartok B , Firestein GS . Fibroblast‐like synoviocytes: key effector cells in rheumatoid arthritis. Immunol Rev. 2010;233(1):233‐255. 20193003 10.1111/j.0105-2896.2009.00859.x PMC2913689

[4] Shinotsuka N , Denk F . Fibroblasts – the neglected cell type in peripheral sensitization and chronic pain?: A systematic view on the current state of the literature. bioRxiv. 2021:2021.2002.2019.431978.

[5] Maes T , Barceló A , Buesa C . Neuron navigator: a human gene family with homology to *unc‐53*, a cell guidance gene from *Caenorhabditis elegans* . Genomics. 2002;80(1):21‐30. 12079279 10.1006/geno.2002.6799

[6] Hu W , Li X , Cheng R , et al. NAV2 facilitates invasion of cutaneous melanoma cells by targeting SNAI2 through the GSK‐3β/β‐catenin pathway. Arch Dermatol Res. 2019;311(5):399‐410. 30997569 10.1007/s00403-019-01909-w

[7] Wang JG , Xu WD , Zhai WT , et al. Disorders in angiogenesis and redox pathways are main factors contributing to the progression of rheumatoid arthritis: a comparative proteomics study. Arthritis Rheum. 2012;64(4):993‐1004. 22006448 10.1002/art.33425

[8] Chakrabarti S , Hore Z , Pattison LA , et al. Sensitization of knee‐innervating sensory neurons by tumor necrosis factor‐α‐activated fibroblast‐like synoviocytes: an in vitro, coculture model of inflammatory pain. Pain. 2020;161(9):2129‐2141. 32332252 10.1097/j.pain.0000000000001890 PMC7431145

