# Supplementary material for: NAV2 positively modulates inflammatory response of fibroblast‐like synoviocytes through activating Wnt/β‐catenin signaling pathway in rheumatoid arthritis
Source: Clin Transl Med. 2021 May 1;11(4):e376. doi: 10.1002/ctm2.376 (PMC8087903; doi:10.1002/ctm2.376)
Supplement: Supplementary file 1 — Supporting Information [file CTM2-11-e376-s001.docx]

**SUPPLEMENTARY INFORMATION**

**NAV2 positively modulates inflammatory response of fibroblast-like synoviocytes through activating Wnt/β-catenin signaling pathway in rheumatoid arthritis**

**Materials and Methods**

**Reagents**

Recombination human TNF-α was purchased from Peprotech (NJ, USA). Complete Freund’s adjuvant (CFA, heat-inactivated Mycobacterium tuberculosis) was obtained from Chondrex (WA, USA). Cell Counting Kit-8 (CCK-8, DOJINDO, Kyushu, Japan). Antibodies were obtained from the following commercial sources: matrix metallopeptidase-3 (MMP-3), MMP-9, Cyclooxygenase-2 (COX-2), Interleukin-6 (IL-6) and glyceraldehyde-3-phosphate dehydrogenase (GAPDH) were purchased from Santa Cruz Biotechnology, Inc. (CA, USA); Glycogen synthase kinase-3β (GSK-3β), β-catenin, cellular-myelocytomatosis viral oncogene (c-myc), Cyclin D1, Vascular cell adhesion molecule-1 (VCAM-1) and Intercellular cell adhesion molecule-1 (ICAM-1) were obtained from Cell Signaling Biotechnology (MA, USA); NAV2 was obtained from Abnova (Taipei, Taiwan); E2F1 was purchased from Abcam (Cambridge, UK).

**Animal model of adjuvant-induced arthritis (AIA) rats**

30 SPF grade Male Sprague-Dawley (SD) rats purchased from Beijing Vital River Laboratory Animal Technology Co. Ltd (Beijing, China) weighing 180-200 g was randomly divided into 2 groups of 15 animals each. The rats were allowed 7 days to adapt to the laboratory environment before experiments were begun. Adjuvant arthritis was induced according to the method described previously.^1^ All animals were under a controlled temperature (23 ± 1°C) and humidity (50% ± 5%) with a 12 h light/dark cycle and free access to the standard food and water. The body weight, hind paw volume, and the arthritis scores were measured by two independent observers on the 0, 10, 15, 20, 25, 30th day after the injection by a plethysmometer. Blood samples were collected from the abdominal aorta of anesthetized rats on day 30 of the experiment. Then, the rats were sacrificed by cervical dislocation. The levels of Interleukin-1 beta (IL-1β), TNF-α, and IL-6 in serum were examined by using commercially available enzyme-linked immunosorbent assay (ELISA) kits according to the manufacturer’s instructions (Multi Sciences, Hangzhou, China). The optical densities were read by using SpectraMax Paradigm (Molecular Devices, CA, USA) at 450 nm. All the experimental processes were conducted within the approved guidelines of the Ethics Review Committee for Animal Research of Macau University of Science & Technology.

**Cell culture and treatment**

Human embryonic kidney (HEK) 293T cells obtained from the American Type Culture Collection were maintained in Dulbecco’s Modification of Eagle’s Medium (DMEM) supplemented with 10% fetal bovine serum (FBS, Gibco, MA, USA) and 1% penicillin-streptomycin (Gibco, MA, USA) at 37℃ in a 95% air/ 5% CO_2_ incubator. FLS are the main effector cells of RA joint synovitis and joint destruction. They can be activated by the pro-inflammatory cytokines IL-1β or TNF-α and secrete a large number of pro-inflammatory mediators. Among them, Nitric oxide synthases (iNOS), IL-6, and COX-2 are classic pro-inflammatory mediators, which play an important role in the occurrence and development of RA.^2^ MH7A cells are an immortal cell line of human FLS constructed by the Japanese Institute of Riken, which can well mimic the pathogenic characteristics of synovial cells *in vitro*. Here we used the MH7A cell line purchased from Guangzhou Jennio Biotech Co., Ltd (Guangzhou, China). The MH7A cells were cultured in DMEM medium, supplemented with 15% FBS and 1% penicillin-streptomycin in a 5% CO_2_ humidified atmosphere at 37 °C. Primary human synovial fibroblast cells were isolated from the synovial tissue using the collagenase digestion method as previously described^1,3^ and cultured in DMEM supplemented with 15% FBS and 1% penicillin-streptomycin at 37℃ in a 95% air/ 5% CO_2_ incubator. As TNF-α is a classical and pivotal inflammatory cytokine known to induce the generation of a range of additional inflammatory cytokines,^4^ we used TNF-α (20 ng/ml) to stimulate MH7A cells to explore the related mechanism in RA.

**Clinical blood samples**

Blood samples were collected from patients with RA and OA, and healthy volunteers under the premise of signing the informed and voluntary consent in the Department of Rheumatology, Longhua Hospital of Shanghai University of Traditional Chinese Medicine (Shanghai, China). All of the epidemiological investigations and classification from volunteers were carried out according to the criteria of the American College of Rheumatology. Clinical blood samples were first separated and extracted white blood cells and then continued to the following experiments. The study was approved by the ethics committee of Longhua Hospital of Shanghai University of Traditional Chinese Medicine. The clinically relevant information and spreadsheet are shown in table S1.

**Histological and immunohistochemical analysis of joint tissue**

For the histologic analysis, rat ankle joint tissues were fixed in 4% buffered formaldehyde, decalcified with 0.5 M ethylenediaminetetraacetic acid (EDTA, pH 8.0), embedded in paraffin, and sectioned at 3 μm thickness. Then the paraffin sections were stained with hematoxylin and eosin (HE). Immunohistochemical analysis was performed on the deparaffinized tissues. The tissue samples were subjected to antigen retrieval by boiling in 10 mM citrate buffer (pH 6.0) for 10 min, treated with 1% H_2_O_2_ for 10 min, and then blocked with 5% bovine serum albumin (BSA) for 1 h at room temperature. The tissue sections were further incubated with primary antibodies at 4°C overnight. After incubation with appropriate secondary antibody for 1.5 h, the sections were visualized by 3, 3-diaminobenidine (DAB). The stained tissue sections were imaged using light microscopy (Leica DM2500, Solms, Germany)

**Micro-computed tomography (Micro-CT) analysis**

At the end of the treatment period, the rats were humanly dispatched, and the left hind paw was amputated and fixed in 4% paraformaldehyde (PFA), then scanned by using *in vivo* micro-CT scanner (SkyScan 1176, Bruker, Belgium). The following scanning parameters were used to obtain high-quality images of the joint of the rat: 53 kV, 470 μA, 65 ms exposure time, 0.7, rotation step in 360°, and a 1 mm Al filter. The images were reconstructed by using NRecon software (Bruker-micro CT, Belgium)

**Small interfering RNA (siRNA) transfection**

Human NAV2 small interfering RNA (commercial si *NAV2*, sc-96275), human E2F1 small interfering (commercial si *E2F1*, sc-29297), and control siRNA (si Scr) were produced by Santa Cruz Biotechnology, Inc. (CA, USA). To introduce siRNA into MH7A cells, the cells were planted on 6-well plates at 30-50% confluence before transfection. Individual siRNA (at 25-50 nM), Lipofectamine RNAiMAX (Invitrogen, CA, USA), and Opti-MEM (Thermo Fisher Scientific, MA, USA) were mixed and incubated at room temperature for 10 min. siRNA Lipofectamine RNAiMAX complexes were added to the cells for 48 h and the medium was replaced by fresh serum DMEM after transfection. Experiments were performed after transfection for 60 h.

**Plasmid constructs and transfection**

E2F1 overexpression vector complementary DNA (cDNA) was constructed by BersinBio Biotech Company (Guangzhou, China) through the insertion of human E2F1 cDNA into the pcDNA3.1 vector. The MH7A cells were transfected with this cDNA constructs with Lipofectamine 3000 (Invitrogen, CA, USA) in compliance with the manufacturer’s protocols. Controls were cells transfected with empty vectors.

**RNA extraction, cDNA synthesis, and real-time quantitative PCR (RT-qPCR)**

Total RNA from pretreated MH7A cells or synovial tissues were extracted by TRIzol Reagent (TaKaRa Biotechnology, Dalian, China). Total RNA (1.0 μg) of each sample was reverse transcribed into cDNA by BIO-RAD cDNA Synthesis Kit (CA, USA) according to the manufacturer’s instructions. The expression of target genes was measured by semi-quantitative qPCR using iTaqTM Universal SYBR Green Supermix (BIO-RAD, CA, USA). RT-qPCR was conducted through ViiA™ 7 Real-Time PCR System (CA, USA) with initial denaturation at 95° C for 10 min and then 40 cycles of denaturation at 95°C for 30 s and annealing at 60° C for 30 s, 72° C for 30 s. GAPDH was used as the reference gene for analyzing the relative target gene expression using the 2^-△△Ct^ method. The primers in the RT-qPCR reaction were used in Table S2.

**Western blot analysis**

Cells and tissue proteins were lysed in RIPA buffer (Cell Signaling Biotechnology, MA, USA) containing protease and phosphatase inhibitor cocktail (Sigma, St Louis, USA). Whole-lysate samples were separated by SDS-PAGE and then were transferred onto nitrocellulose membranes for immunoblot analysis. The membranes were blocked with 5% non-fat milk in Tris-buffered saline with Tween 20 (TBST) for 1 h at room temperature in a rotating platform and then incubated with the indicated primary antibodies overnight at 4℃. The membrane was washed 3 times in TBST for 5 min each in a rotating platform and then incubated with an anti-rabbit or mouse secondary antibody for 1 h at room temperature in a rotating platform. Finally, the membrane was washed 3 times with TBST and all the bands in membranes were imaged by GE Amersham Imager 600 machine (IL, USA) and the intensities of bands were analyzed by ImageJ software (MD, USA). GAPDH was considered as a loading control.

**Immunofluorescence staining analysis**

MH7A cells were seeded on glass coverslips placed in 6-well plates. Following different treatments, cells were fixed with 4% paraformaldehyde for 20 min, followed by permeabilization with 0.3% Triton X-100 in PBS for 10 min. And then the slides were blocked in PBS with 1% BSA for 1 h at room temperature and incubated overnight with primary antibodies at 4°C. Appropriate secondary antibodies were added and incubated for 2 h at room temperature. The nuclei were stained with DAPI for 5 min. The images were captured using a fluorescence microscope (Olympus IX73, Tokyo, Japan).

**MH7A cells proliferation assay**

MH7A cells were seeded in 96-well plates at a density of 5.0×10^3^ cells/ well and cultured overnight for cell adhesion. The cells were treated with siRNA negative or si NAV2 for 48 h and then stimulated with TNF-α for 12 h. The CCK-8 assay was utilized to determine cell proliferation according to the manufacture’s instruction. The absorption value was measured at 450 nm using SpectraMax Paradigm (Molecular Device, CA, USA). All of the values were standardized by comparison with the data from the untreated cells.

**MH7A cells invasion assay**

Following the transfection with the siRNA for NAV2 or the control siRNA for 48 h, the MH7A cells were subjected to an invasion assay. For the *in vitro* invasion assay, Matrigel basement membrane matrix (BD Biosciences, Oxford, UK) was pre-coated on the membrane of the upper chamber and a total of 5×10^4^ cells were re-suspended in 200 μl of medium with 1% FBS and seeded in the upper chamber, while the lower chamber was filled with complete medium. Cells were seeded as a migration assay according to our previously described method.^1^ Finally, the stained cells were counted as the mean number of cells per 6 random fields for each assay. All of the experiments were replicated 3 times.

**Wound Healing Assay**

MH7A cells were planted into a 6-well culture plate and grown to confluence up to about 60%. And then cells were serum-deprived and treated with NAV2-siRNA. And then the medium was replaced with fresh DMEM within TNF-α (20 ng/ml) stimulation. The plate was then scratched with a sterile plastic pipette tip and washed with PBS twice to remove deciduous cells. There was a single wound was created in the center of the cell monolayer. After 12 h simulation, the wound areas were respectively photographed using a microscope (Olympus IX73, Tokyo, Japan) equipped with a digital camera, and three assays of wound area were made at random fields. The extent of wound closure was presented as the percentage by which the original scratch area had decreased at each measured time point. The data are obtained from three independent experiments.

**Luciferase reporter assay**

A fragment containing the core promoter region of NAV2 (-2000 - +500) was inserted between the XhoI and HindІІІ sites of the firefly luciferase vector pGL4.10 (Promega, WI, USA). The NAV2 promoter-luciferase reporter plasmids were constructed by BersinBio (Guangzhou, China). Cultured 293T cells were transfected with either of these plasmids (together with a plasmid containing the Renilla luciferase gene to serve as a reference for transfection efficiency), with or without co-transfection of the plasmid to overexpression of E2F1. After 48 h transfection and stimulation by TNF-α for 12 h, the firefly luciferase activity and Renilla luciferase activity were measured on a luminometer (Berthold Technologies, Germany).

**Chromatin immunoprecipitation (CHIP) assay**

CHIP assay was performed by using CHIP Kit (Bes5001, BersinBio, Guangzhou, China) according to the manufacturer’s instructions. Briefly, the cells were fixed with 1% formaldehyde, and the cross-linking was quenched by adding in 100 μl of 1.375 M glycine per milliliter of culture. The samples were sonicated on ice to shear the DNA into 200 to 600 bp fragments. For each total cell lysate, one third was used as the DNA input control, another third was immunoprecipitated with anti-E2F1 antibodies, and the last third was subjected to non-immune rabbit IgG (Cell Signaling Biotechnology, MA, USA). DNA fragments were purified by spin columns (Qiagen, Hilden, Germany), and RT-qPCR was performed to amplify the segment in the promoter region of NAV2 with the following primers:

**Prime 1**, Forward: 5’-TGCCATCCCCTCATCCTACA-3’

Reverse: 5’-CACACACCAAAGCAGAAGGC-3’

**Prime 2**, Forward: 5’-AGCACAAGCCTCCCAGATTC-3’

Reverse: 5’-ACGCAGAACAAGGATCCCTG-3’

**Prime 3**, Forward: 5’- GCGTTAGCAGGCATGATTGG-3’

Reverse: 5’- TCTCGCACTGCACTCCGG-3’

**Statistical analysis**

The statistical analysis was performed using GraphPad Prism version 8.0. All of the values are expressed as the mean ± SEM. The two-sample *t*-test for comparing the means of two groups, and the one-way ANOVA with Dunnett’s multiple comparisons for comparing means among multiple groups. The difference is considered statistically significant at the *P < 0.05* level.

REFERENCES

1. Wu WJ, Jia WW, Liu XH, et al. S-propargyl-cysteine attenuates inflammatory response in rheumatoid arthritis by modulating the Nrf2-ARE signaling pathway. *Redox Biol*. 2016; 10:157-167.

2. Jin JH, Kim JS, Kang SS, Son KH, Chang HW, Kim HP. Anti-inflammatory and anti-arthritic activity of total flavonoids of the roots of Sophora flavescens. *J Ethnopharmacol.* 2010;127(3):589-595.

3. Wu W, Qin M, Jia W, et al. Cystathionine-gamma-lyase ameliorates the histone demethylase JMJD3-mediated autoimmune response in rheumatoid arthritis. *Cell Mol Immunol.* 2019;16(8):694-705.

4. Mukaida N, Mahe Y, Matsushima K. Cooperative interaction of nuclear factor-kappa B- and cis-regulatory enhancer binding protein-like factor binding elements in activating the interleukin-8 gene by pro-inflammatory cytokines. *J Biol Chem.* 1990; 265(34):21128-21133.


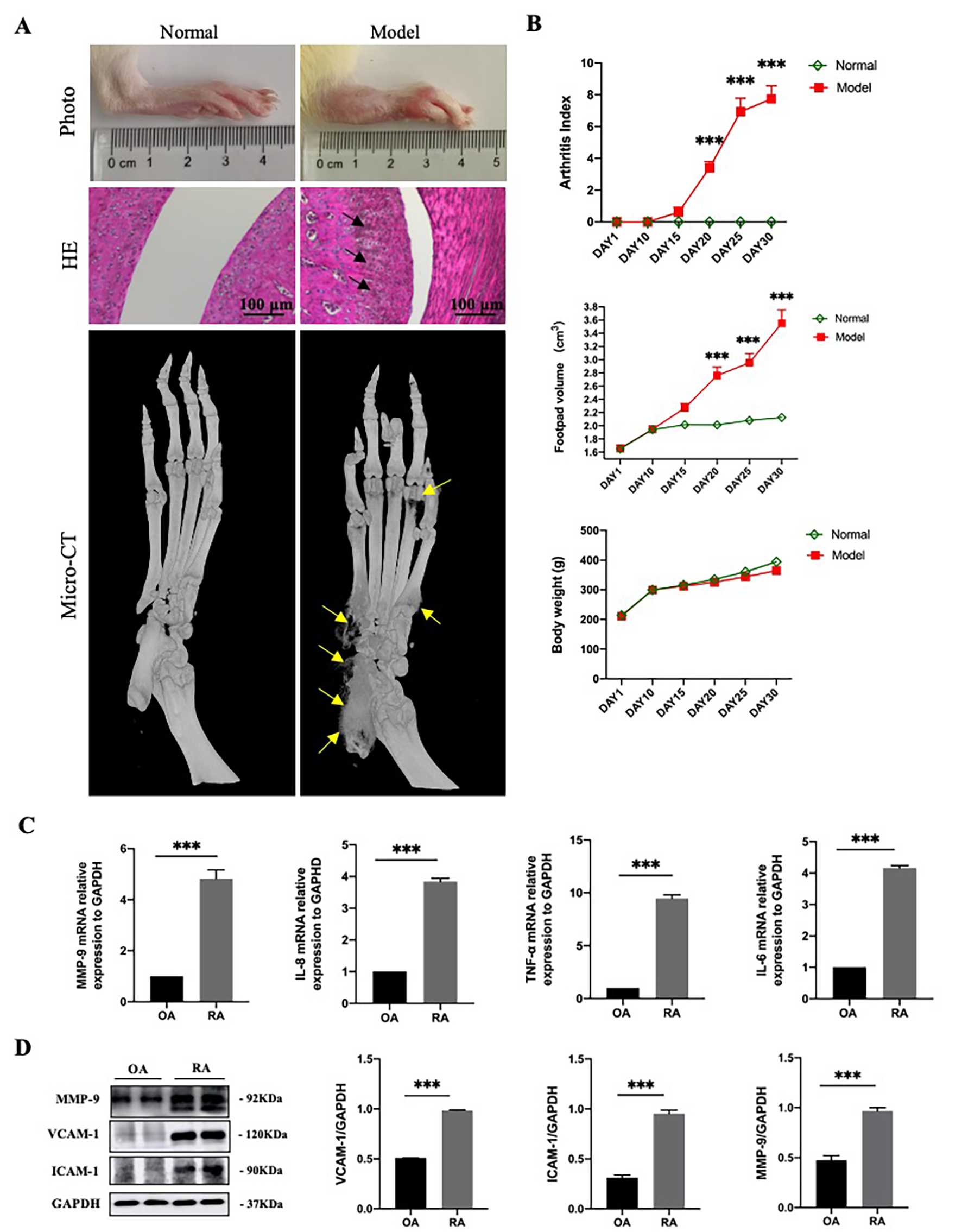


Figure S1. Macroscopic evidence of arthritis, such as erythema or swelling, was markedly observed in the AIA rats, Micro-CT images of ankles and H&E staining analysis of representative knee sections from the normal and AIA rats at day 30 after immunization were shown in A. B, Arthritis index, increased hind paw volume and body weights of rats were measured (n = 15 for each group). The expression of inflammatory mediators was increased in FLS from RA patients. C, Expression of *MMP-9*, *IL-8*, *TNF-α*, and *IL-6* mRNA in FLS. D, The protein levels of MMP-9, VCAM-1, and ICAM-1 were examined. Data are means ± SEM from at least 3 independent experiments. *** *P* < 0.001.

**
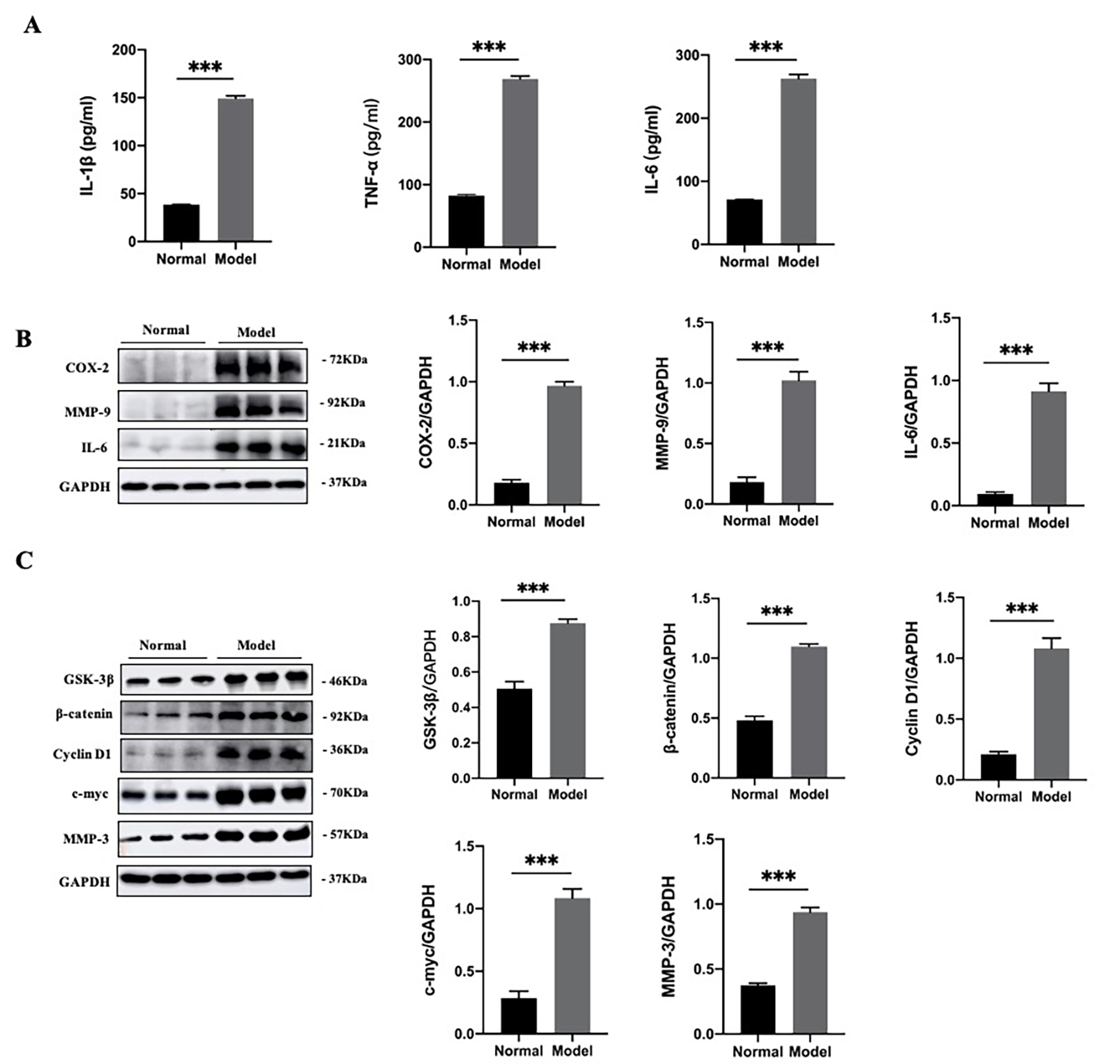
**

Figure S2. The expression of inflammatory mediators and related proteins in the Wnt/β-catenin pathway were increased in AIA rats. A, The levels of serum IL-1β, TNF-α and IL-6 of AIA rats measured by ELISA. B, Western blot results and quantitative analysis of COX-2, MMP-9, IL-6, expression in synovial tissues from AIA rats were shown. C, Western blot results and quantitative analysis of GSK-3β, β-catenin, c-myc, CyclinD1 and MMP-3 expression in synovial tissues from AIA rats were shown. Data are presented as mean ± SEM of more than 3 independent experiments. *** *P* < 0.001

**
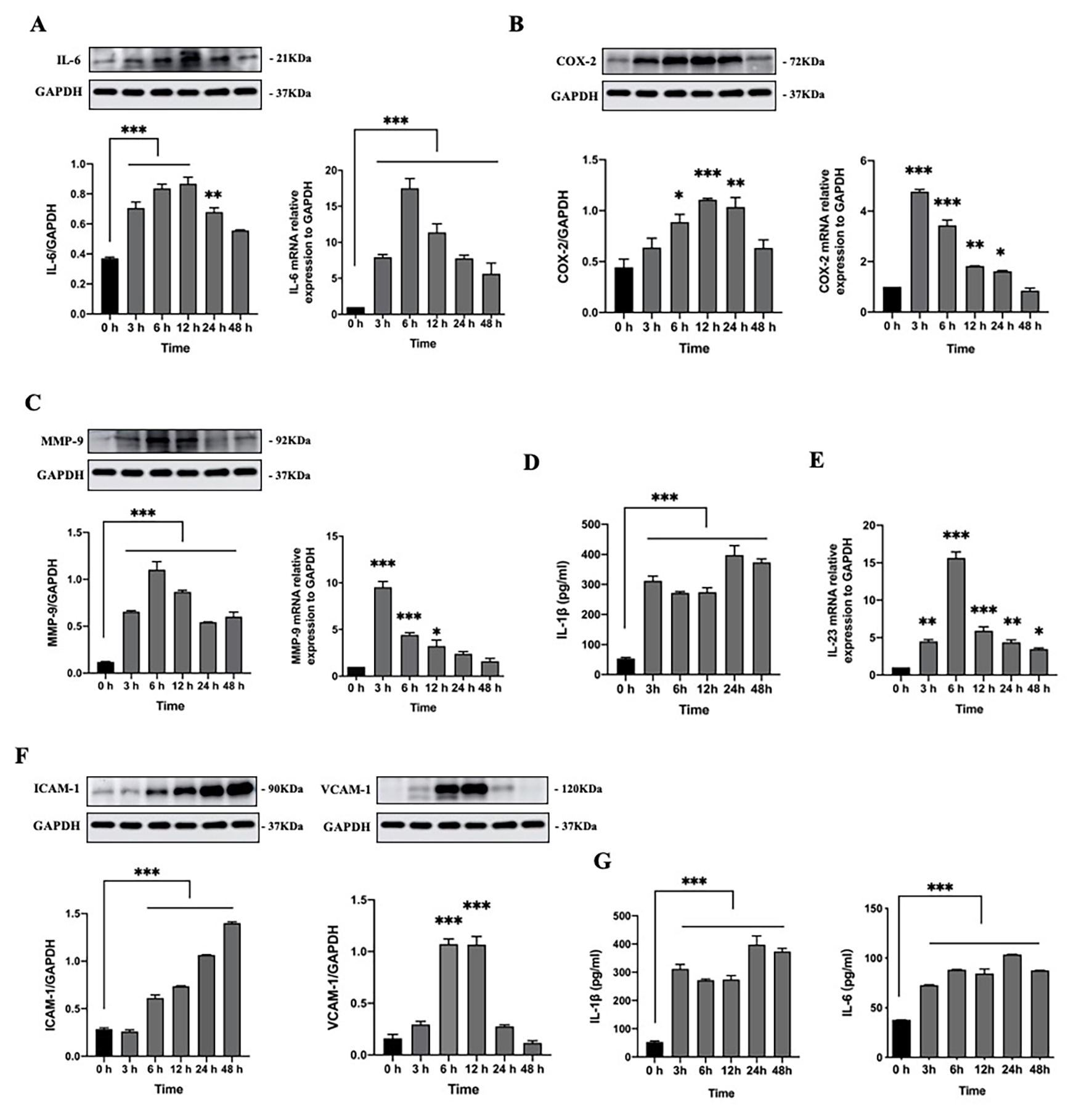
**

Figure S3. Upregulation of RA-associated inflammatory mediators in MH7A cells stimulated with TNF-α. MH7A cells were stimulated with or without TNF-α (20 ng/ml) for the indicated periods, the inflammatory mediator levels were analyzed as described in Materials and Methods, respectively. A-C, IL-6, COX-2 and MMP-9 mRNA and protein expression, GAPDH was used as loading control. D and E, *IL-1β*, *IL-23* mRNA expression. F, ICAM-1 and VCAM-1 protein expression. G, IL-1β and IL-6 production in the culture supernatants. Data are presented as mean ± SEM of more than 3 independent experiments. * *P* < 0.05, ** *P* < 0.01, *** *P* < 0.001 vs. unstimulated cells.


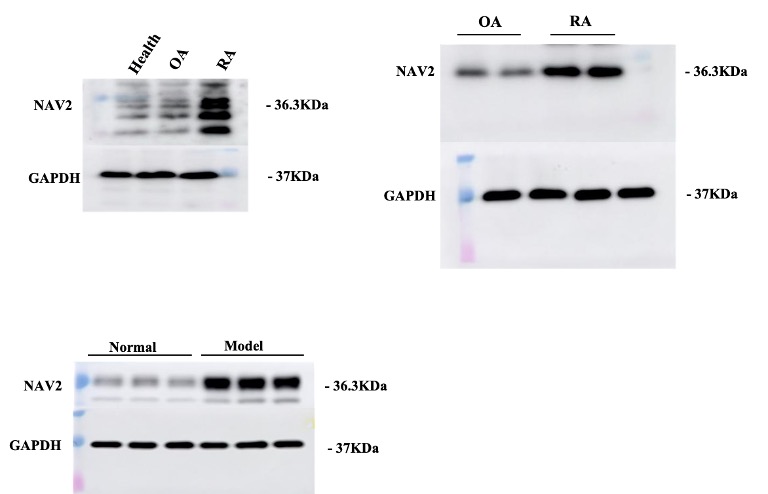


Figure S4. Untrimmed gel images with molecular weight ladders correspond to Figure 1.

**
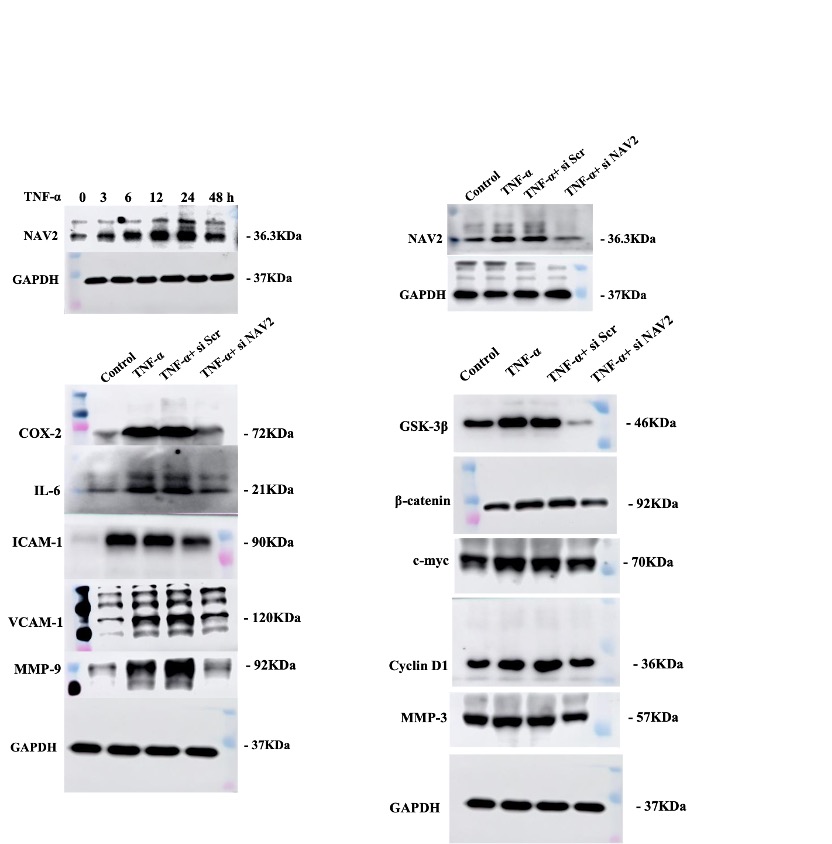
**

Figure S5. Untrimmed gel images with molecular weight ladders correspond to Figure 2

**
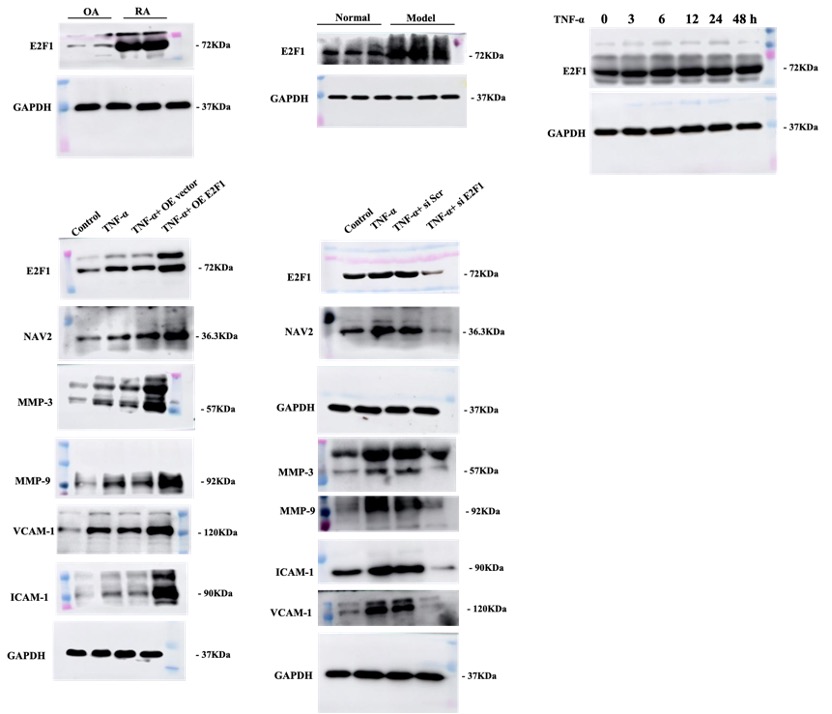
**

Figure S6. Untrimmed gel images with molecular weight ladders correspond to Figure 3

**
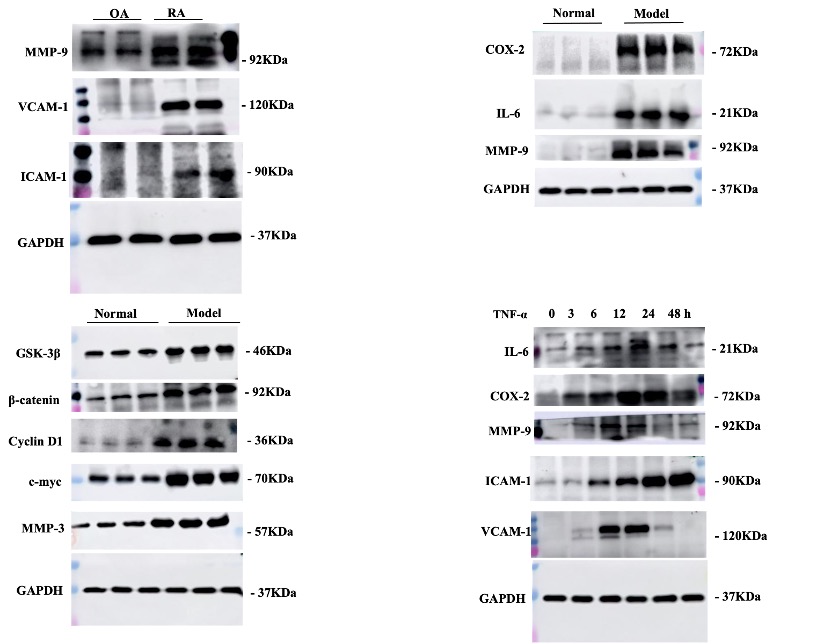
**

Figure S6. Untrimmed gel images with molecular weight ladders correspond to Figure S1, Figure S2 and Figure S3

**Table S1:** RA, OA and healthy volunteers baseline datasheet

|  | **Parameter** | **RA (N=****20)** | **OA (N=9)** | **Healthy (N=6)** |
| --- | --- | --- | --- | --- |
|  |  |  |  |  |
|  | **Age (year (mean (range)))** | 61(37~79) | 56(58~75) | 46(18~65) |
|  | **Gender (N(F/M))** | 18/2 | 7/2 | 3/3 |
|  | **RF (>20 IU/mL)** | 16/20 | NA | NA |
|  |  |  |  |  |

RF, Rheumatoid factors; NA, Not assessed.

**Table S2：** Primers used for RT-qPCR validation

| Gene name | Primer name | Primer sequence (5’ to 3’) |
| --- | --- | --- |
| NAV2 | Homo _NAV2_F | GAGGGACGGGAGTTGACAGA |
|  | Homo _NAV2_R | CAGTTGAGCAGCCCATTGAA |
| E2F1 | Homo _E2F1_F | ACGCTATGAGACCTCACTGAA |
|  | Homo _E2F1_R | TCCTGGGTCAACCCCTCAAG |
| GAPDH | Homo _GAPDH_F | GATTCCACCCATGGCAAATTCC |
|  | Homo _GAPDH_R | GCATCGCCCCACTTGATTTT |
| MMP-9 | Homo _MMP-9_ F | TTTGAGTCCGGTGGACGATG |
|  | Homo _MMP-9_ R | GCTCCTCAAAGACCGAGTCC |
| IL-8 | Homo _IL-8_ F | ACACTGCGCCAACACAGAAA |
|  | Homo _IL-8_ R | GTTTTCCTTGGGGTCCAGACA |
| TNF-α | Homo _TNF-α_ F | CGAGCTGGGTTTCTTTATACCG |
|  | Homo _TNF-α_R | GCAATTTGGGGATATTCTCCTGT |
| IL-6 | Homo _IL-6_ F | CAGCGACACGCCGACTATAC |
|  | Homo _IL-6_ R | CCTCCAAGGCATTTCCTCTTTT |
| COX-2 | Homo _COX-2_ F | GCATTCTTTGCCCAGCACT |
|  | Homo _COX-2_ R | AAAGGCGCAGTTTACGCTGT |
| IL-23 | Homo _IL-23_ F | TGTGGAGATGGCTGTGAC |
|  | Homo _IL-23_ R | GAAGCGGAGAAGGAGACG |
| NAV2 | Rat _NAV2_ F | GGCTACAGACCTACGCATCC |
|  | Rat _NAV2_ R | GAAGGAGCTGCGTAACCAGT |
| GAPDH | Rat _GAPDH_ F | CTTCTCTTGTGACAAAGTGGACAT |
|  | Rat _GAPDH_ R | TTCTCAGCCTTGACTGTGCC |
